# Low level of swiprosin-1/EFhd2 in vestibular nuclei of spontaneously hypersensitive motion sickness mice

**DOI:** 10.1038/srep40986

**Published:** 2017-01-27

**Authors:** Zhi-Bin Wang, Ping Han, Ling-Chang Tong, Yi Luo, Wei-Heng Su, Xin Wei, Xu-Hong Yu, Wei-Ye Liu, Xiu-Hua Zhang, Hong Lei, Zhen-Zhen Li, Fang Wang, Jian-Guo Chen, Tong-Hui Ma, Ding-Feng Su, Ling Li

**Affiliations:** 1 Department of Pharmacology, College of Pharmacy, Second Military Medical University, Shanghai 200433, China; 2 Department of Pharmacology, Tongji Medical College, Huazhong University of Science and Technology, Wuhan, Hubei 430030, China; 3 Basal medical College, Dalian Medical University, Dalian, Liaoning 130041, China

## Abstract

Susceptibility to motion sickness (MS) varies considerably among humans. However, the cause of such variation is unclear. Here, we used a classical genetic approach to obtain mouse strains highly sensitive and resistant to MS (SMS and RMS). Proteomics analysis revealed substantially lower swiprosin-1 expression in SMS mouse brains. Inducing MS via rotary stimulation decreased swiprosin-1 in the mouse brains. Swiprosin-1 knockout mice were much more sensitive to motion disturbance. Immunohistochemistry revealed strong swiprosin-1 expression in the vestibular nuclei (VN). Over-expressing swiprosin-1 in the VN of SMS mice decreased MS susceptibility. Down-regulating swiprosin-1 in the VN of RMS mice by RNAi increased MS susceptibility. Additional *in vivo* experiments revealed decreased swiprosin-1 expression by glutamate via the NMDA receptor. Glutamate increased neuronal excitability in SMS or swiprosin-1 knockout mice more prominently than in RMS or wild-type mice. These results indicate that swiprosin-1 in the VN is a critical determinant of the susceptibility to MS.

Motion sickness (MS) refers to a series of central and neuro-vegetative responses to the perception of motion, whether real or apparent^1^,^2^,^3^. Depending on the cause, MS is also referred to as sea sickness, car sickness, air sickness, space sickness or simulation sickness^4^,^5^. The symptoms typically follow a sequence from stomach discomfort and nausea to dizziness and vomiting^6^. Subjects with severe MS may even develop dehydration and electrolyte disturbances^7^,^8^. MS is a common problem, and almost anyone with a functional vestibular system can develop MS.

The vestibular system is critical for MS to occur. The primary functions of the vestibular system include spatial orientation and maintenance of balance^4^. Subjects with non-functioning labyrinths are immune to MS^9^. Bilateral vestibular neurectomy or labyrinthine ablation causes susceptible laboratory animals to become immune to MS^3^,^10^. In contrast, electrical vestibular stimulation can induce MS-like symptoms in human subjects^11^. The anatomy and function of the vestibular system have been well studied. First, hair cells with polarization vectors in the crista ampullaris membrane of the semicircular canals and the basilar membrane of the otolithic macula detect endolymph fluid motion relative to the bony structure and convert the mechanical motion to electrical signals^12^. Next, the vestibular nerve transmits electrical activity from the hair cells to the central vestibular nuclei (VN) of the brainstem. Vestibular afferents are active even at rest and are strikingly sensitive to head acceleration^13^,^14^. Finally, the VN processes vestibular afferent information and contacts with multiple nuclei and brain regions to form multiple vestibular nerve pathways^13^. Glutamate and acetylcholine are the main excitatory neurotransmitters both in vestibular afferent nerves and in the VN for transmitting excitatory nerve impulses^15^,^16^,^17^.

In contrast to our understanding of the anatomy involved in MS, the molecular mechanisms underlying MS development and the regulation of MS remain largely unknown. The development of anti-MS drugs are thus hindered. Scopolamine and dimenhydrinate are the main anti-MS drugs currently available. However, both agents are highly sedative due to general depression of the central nervous system.

Susceptibility to MS varies considerably across individuals^4^,^18^. It is expected that an in-depth understanding of the susceptibility to MS will be helpful in developing more specific treatments for MS. Abe *et al*. found that parents who are susceptible to MS are more likely to have children with higher sensitivities to MS. Moreover, twin studies demonstrated familial aggregation and significant heritability^19^,^20^. These findings highlight the importance of genetic factors in determining susceptibility to MS. In this regard, creating an animal model in which genetic factors are involved in MS would be of great value. The goal of the current study was to establish a genetic animal model that is hypersensitive to MS and to explore the possible factors governing MS susceptibility.

Mice lack a vomiting reflex^21^ but develop other symptoms typical of MS upon rotation: piloerection, tremble, and urinal and faecal incontinence. In a previous study, we used a score based on the symptoms mentioned above as an index to reflect the severity of MS in mice^22^. In the current study, we used selective breeding to obtain two mouse strains that were highly sensitive to MS (SMS) and resistant to MS (RMS). Proteomics studies revealed decreased expression of swiprosin-1, a protein selectively expressed in VN neurons, in SMS mice.

In the next set of experiments, we considered wild-type mice to further study swiprosin-1. The results demonstrated that swiprosin-1 level changes are specific in MS. Additionally, we found that swiprosin-1 is an anti-MS protein.

## Results

### Generation of RMS and SMS mice

**Figure 1:**
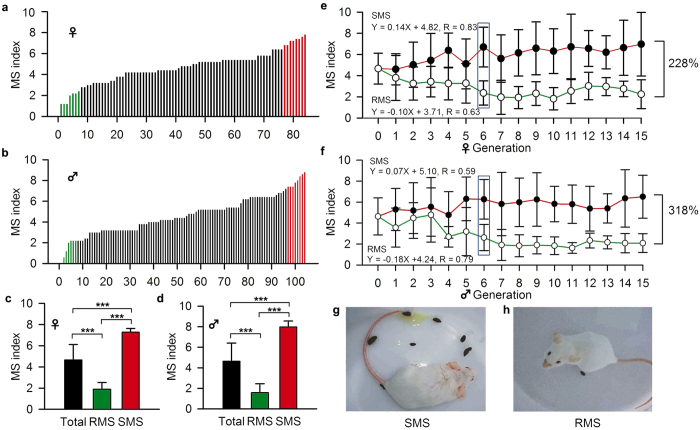
Establishment of two stable mouse strains spontaneously sensitive and resistant to MS. (**a**,**b**) The MS index in response to rotary stimulation for normal female and male Kunming mice. (**c**,**d**) The MS index in response to rotary stimulation for female and male mice highly sensitive (SMS) or resistant (RMS) to MS. (**e**,**f**) Separation of inherited strains that were SMS or RMS after fifteen generations of breeding guided by the MS phenotype. (**g**,**h**) Response of the sensitive (*left*) and resistant (*right*) strains to rotary stimulation-induced MS. The data are quantified as the mean ± SD. ***p < 0.001 compared to control or as indicated.

In a previous study conducted in our laboratory, we noticed significant variations in the susceptibility to MS in mice, and the MS index was normally distributed^22^. To unravel the genetic basis of the varying susceptibility to MS, we obtained SMS and RMS strains by phenotype-guided breeding using genetically heterogeneous outbred mice as the founders (Fig. 1a,b). The MS index of the founders varied from 0 to 8.8 and did not differ between the female and male subjects (4.7 ± 1.5 vs. 4.6 ± 1.8, respectively) (Fig. 1c,d). The F0 generation included eight pairs of mice with low (green symbols) and high (red symbols) MS indexes (<2.0 vs. >7.0, respectively).

MS susceptibility stably separated after selective breeding for six generations (Fig. 1e,f, blue rectangles). At the 15^th^ generation, the MS index was 7.0 ± 3.0 in female SMS mice and 2.2 ± 1.4 in female RMS mice, with a difference of 228% (Fig. 1e). The MS index was 6.5 ± 2.1 in male SMS mice and 2.1 ± 0.9 in male RMS mice for a difference of 318% (Fig. 1f). The symptoms of MS after 40-min rotary stimulation were much more severe in the SMS mice than in the RMS mice (Fig. 1g,h). Thus, we successfully established two stable mouse strains: mice hypersensitive to MS and mice hyposensitive to MS.

### Characteristics of SMS mice

**Figure 2:**
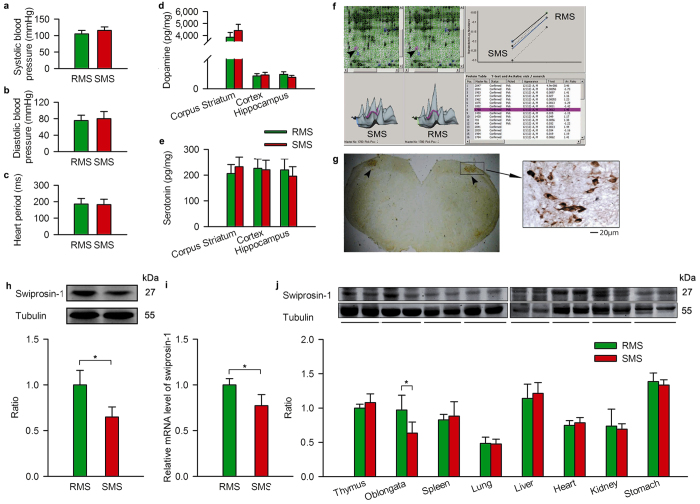
Different expression of swiprosin-1 in the VN of RMS and SMS mice. (**a–c**) Systolic blood pressure, diastolic blood pressure and heart period of RMS and SMS mice. (**d**,**e**) Dopamine and serotonin content in the corpus striatum, the cerebral cortex and the hippocampus of RMS and SMS mice. (**f**) Differential swiprosin-1 levels were identified between the brains of RMS and SMS mice by two-dimensional fluorescence difference gel electrophoresis analysis. (**g**) Localization of swiprosin-1 in the VN of the medulla oblongata of the mice by immunohistochemistry. (**h**) Differential swiprosin-1 level was confirmed in the brains between the RMS and SMS mice by Western blotting. (**i**) Swiprosin-1 in different tissues of the RMS and SMS mice. Data are quantified as the mean ± SD. *p < 0.05 compared to control or as indicated; n = 3–8 in each group.

Because the differences in MS index between the RMS and SMS mice were significant and persistent, mice from the 11^th^ generation were used for subsequent experiments. It is well known that the vestibular-autonomic nervous system and its control over the cardiovascular system and brain monoamines are implicated in MS^23^,^24^,^25^. We measured systolic blood pressure, diastolic blood pressure and the heart period (Fig. 2a,b) and determined the concentration of dopamine and serotonin in the cerebral cortex, the corpus striatum and the hippocampus (Fig. 2d,e), but we found no differences between the SMS and RMS mice.

To identify candidate proteins responsible for susceptibility to MS, we carried out a proteomics analysis of the brain in SMS *vs*. RMS mice. Mass spectrometry followed by bioinformatics analysis of cytosolic proteins in the brain of SMS *vs*. RMS revealed the following five differentially expressed proteins: swiprosin-1, aspartate-β-hydroxylase, mKIAA0106, glycolysis phosphoglycerate mutase 1 and peroxiredoxin-6 (Fig. 2f, Supplement 1). The differential expression of swiprosin-1 was most prominent as confirmed using Western blot and RT-PCR (Fig. 2h,i).

Swiprosin-1 was first identified in human lymphocytes, predominantly in CD^8+^ lymphocytes^26^ and later in immature, resting or activated B cells^27^,^28^, mast cells^29^,^30^, human peripheral blood mononuclear cells^31^ and mouse platelets^32^. Swiprosin-1 was also found in non-lymphoid tissue, especially in the brain^33^,^34^,^35^. Autonomic function is primarily controlled by the medulla oblongata in the lower brainstem^36^. Immunohistochemistry revealed strong swiprosin-1 expression in the VN (Fig. 2g), a brain region critical for MS^37^. Movement sensed by the semicircular canals and otolith organs is transmitted to the vestibular sensory cells (hair cells), which in turn transmit electrical signals via the VN to the oculomotor control areas, the cerebellum, and the spinal cord^14^. Swiprosin-1 expression did not differ between the SMS and RMS mice in the liver, heart, kidneys, stomach, thymus, spleen and lungs (Fig. 2j), suggesting the specificity of altered expression in the VN.

### Selective response of swiprosin-1 to motion stimulus

**Figure 3:**
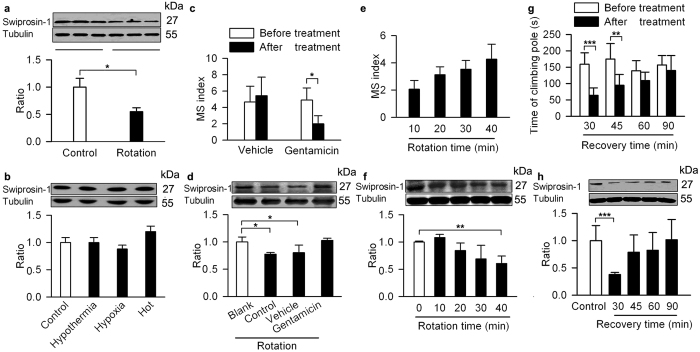
Selective response of swiprosin-1 to motion stimulus. Protein levels of swiprosin-1 were determined by Western blotting of the VN of mice that underwent with rotary stimulation (**a**), hypoxia, hypothermia and hyperthermia (**b**). The MS index (**c**) and swiprosin-1 level in the VN (**d**) after intratympanic injection of gentamicin and after undergoing rotary stimulation. The MS index (**e**) and swiprosin-1 levels in the VN (**f**) after rotation for different stimulation times. (**g**) Time of pole climbing at different time points after rotary stimulation. Behavioural recovery coincided with swiprosin-1 levels at different time point (**h**). Data quantified as the mean ± SD. *p < 0.05, **p < 0.01, ***p < 0.001 compared to control or as indicated; n = 3–6 in each group.

To determine the change in swiprosin-1 after rotatory stimulus and the specificity of this change, we examined swiprosin-1 expression after rotation and under hypoxia, hypothermia and heat conditions. The result showed down-regulated swiprosin-1 in the VN after a 40-min rotation session (Fig. 3a) but not after other types of stressors, indicating that the swiprosin-1 response is specific to motion stimulus (Fig. 3b). Bilateral labyrinthectomy that eliminates vestibular afferent function prevents MS^38^. Gentamicin is an agent widely used for chemical labyrinthectomy^39^. Histological results showed that hair cells and Sertoli cells were nearly absent after chemical labyrinthectomy (Supplement 2). In our experiments, the mouse MS index was decreased 10 days after intratympanic injection of gentamicin (Fig. 3c). The down-regulation of swiprosin-1 after rotary stimulation was not seen after labyrinthectomy (Fig. 3d), suggesting that the response of swiprosin-1 to rotary stimulus requires the integrity of the vestibular system.

We noticed a temporal relationship between the swiprosin-1 level and the MS index along the development and dissipation of rotation-induced MS: the swiprosin-1 level gradually decreased, whereas the MS index gradually increased with prolonged rotation time (Fig. 3e,f). After the session, recovery of swiprosin-1 expression coincided with motor coordination recovery, as reflected by a pole-climbing test (Fig. 3g,h).

### Swiprosin-1 influences the MS index

**Figure 4:**
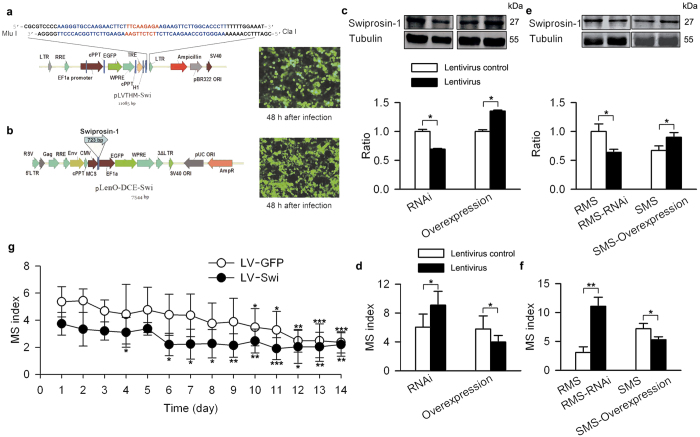
Contribution of swiprosin-1 level in the VN on mouse MS index. (**a**) Schematic diagram of LV-shRNA-Swi (*left*) and infected 293 T cells (*right*). An interference sequence was inserted between the *Mlu* I and *Cla* I restriction sites and confirmed by DNA sequencing. (**b**) Schematic diagram of LV-Swi (*left*) and infected 293 T cells (*right*). Swiprosin-1 full-length coding sequence was inserted between the *BamH* I and *Sal* I restriction sites and confirmed by DNA sequencing. Swiprosin-1 level in the VN after injection of LV-shRNA-Swi or LV-Swi into the VN of Kunming mice (**c**) and RMS and SMS mice (**e**). MS index after injection of LV-shRNA-Swi or LV-Swi into the VN of Kunming mice (**d**) and RMS and SMS mice (**f**). (**g**) MS index after injection of LV-GFP or LV-Swi in the VN of Kunming mice during the adaption period. Data were quantified as the mean ± SD. *p < 0.05, **p < 0.01, ***p < 0.001 compared to control or as indicated; n = 6–8 in each group.

To further examine the function of swiprosin-1 in the VN, we constructed lentiviruses expressing siRNA of *swiprosin-1* (LV-shRNA-Swi) or full-length *swiprosin-1* (LV-Swi) (Fig. 4a,b). In wild-type control mice, stereotactic injection with LV-shRNA-Swi reduced the level of swiprosin-1 in the VN (Fig. 4c, left) and increased the MS index (Fig. 4d, left). Injection of LV-Swi into the VN (Fig. 4c, right) significantly decreased the MS index (Fig. 4d, right). Reducing swiprosin-1 in the VN of RMS mice (Fig. 4e, left) increased the MS index (Fig. 4f, left). Over-expressing swiprosin-1 in the VN of SMS mice (Fig. 4e, right) decreased the MS index (Fig. 4f, right). Habituation is a common characteristic of MS^40^. Repeated exposure to motion decreases susceptibility to MS. In the current study, over-expressing swiprosin-1 in the VN decreased the duration required for habituation from 10 to 6 days (Fig. 4g).

**Figure 5:**
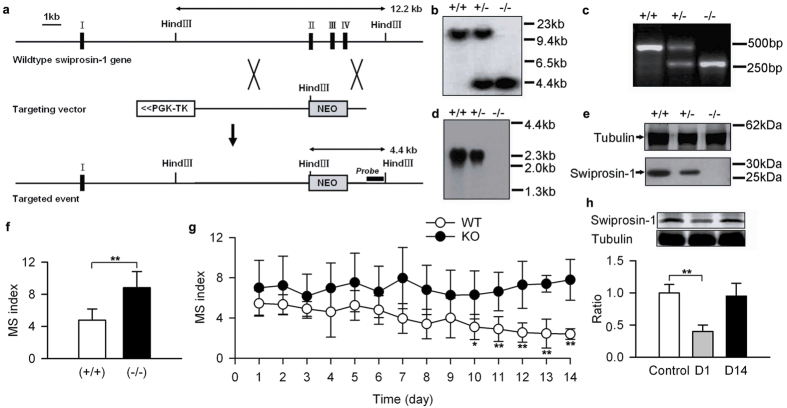
MS in swiprosin-1 knockout mice. (**a**) Gene targeting construct and endogenous *swiprosin-1* locus. (**b**) Southern blot analysis of Hind3-digested mouse liver genomic DNA confirming the properly targeted *swiprosin-1* gene locus. Probes used for Southern blotting analysis are shown by thick black bars in (**a**) along with the expected sizes of hybridized fragments, as indicated. (**c**) PCR genotyping of the *swiprosin-1* allele. Predicted sizes of wild-type and null bands are 462 bp and 263 bp, respectively. (**d**) Northern blot of mouse brain probed with a 500-bp fragment of the mouse *swiprosin-1* 3′ coding sequence. (**e**) Immunoblots of mouse brains using tubulin and swiprosin-1 antibodies. +/+, wild-type; +/−, heterozygous; and −/−, Swiprosin-1 null. (**f**) MS index of wild-type (*swiprosin-1*^+/+^) and homozygous (*swiprosin-1*^−/−^) mice. (**g**) MS index of wild-type and swiprosin-1 knockout mice during the adaption period. (**h**) Swiprosin-1 level in the VN of wild-type mice on the first day and 14^th^ day of the adaption period. Data were quantified as the mean ± SD. *p < 0.05, **p < 0.01 compared to control or as indicated; n = 6–8 in each group.

To further determine the function of swiprosin-1 in the susceptibility of MS, we generated mice lacking swiprosin-1 (*swiprosin-1*^−/−^ mice; generated in this laboratory; Fig. 5a–e). *Swiprosin-1*^−/−^ mice were more sensitive to rotation in comparison to wild-type mice (Fig. 5f). The MS index tapered to a stable level upon daily exposure to rotation over 10–14 days (Fig. 5g) in the wild-type control mice but not in the *swiprosin-1*^−/−^ mice (Fig. 5g) or in the SMS mice (Supplement 3). In the VN of the *swiprosin-1*^+/+^ mice, swiprosin-1 recovered to the baseline level by day 14 (Fig. 5h), suggesting that the swiprosin-1 level in the VN is closely associated with habituation to MS. Additionally, we also verified the distribution and function of swiprosin-1 in cynomolgus monkeys, and the results were similar to that observed in mice (Supplement 4).

### Glutamate down-regulates swiprosin-1

**Figure 6:**
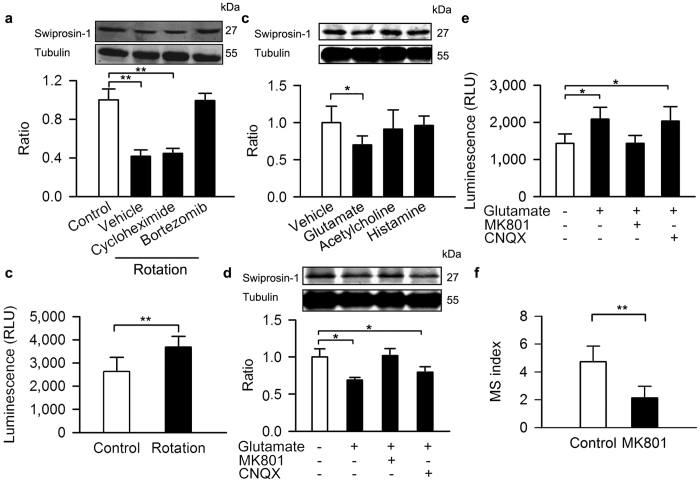
Glutamate down-regulates swiprosin-1 protein level *in vivo*. (**a**) Swiprosin-1 level in the VN after injection of bortezomib and cycloheximide in the VN of wild-type control mice after rotary stimulation. (**b**) Chymotrypsin-like protease activity of the VN after rotary stimulation. (**c**) Swiprosin-1 level in the VN after injection of neurotransmitters into the VN of wild-type control mice. Swiprosin-1 level (**d**) and chymotrypsin-like protease activity (**e**) in the VN after injection of glutamate, MK801 or CNQX into the VN of wild-type control mice. (**f**) MS index after injection of MK801 into the VN of wild-type control mice. Data were quantified as the mean ± SD. *p < 0.05, **p < 0.01 compared to control or as indicated; n = 6–8 in each group.

Decreased swiprosin-1 expression in response to rotation was prevented by prior injection of bortezomib into the VN to block proteasome-dependent protein degradation but not by injection of cycloheximide, which inhibits protein synthesis (Fig. 6a). We also found increased chymotrypsin-like protease activity after rotary stimulus (Fig. 6b), suggesting that the rapid decrease of swiprosin-1 is due to increased degradation but not reduced protein synthesis.

The activity of VN neurons could be influenced by many neurotransmitters, including acetylcholine, glutamate, and histamine^41^,^42^. When these transmitters were applied *in vivo* to the VN in our experiments, only glutamate decreased swiprosin-1 (Fig. 6c). Glutamate is the main excitatory neurotransmitter of vestibular nerve afferents^43^. Vestibular afferent nerves release considerable amounts of glutamate to the vestibular nucleus upon stimulation^44^. *In vivo* experiments showed that the NMDA receptor antagonist MK801 could prevent the reduction of swiprosin-1 levels and the chymotrypsin-like protease activity increase in the VN of mice after rotary stimulation (Fig. 6d,e). Stereotactic injection of MK801 into the VN also lowered the MS index of the mice (Fig. 6f).

### Swiprosin-1 deficiency increases glutamate-induced excitation of the VN

**Figure 7:**
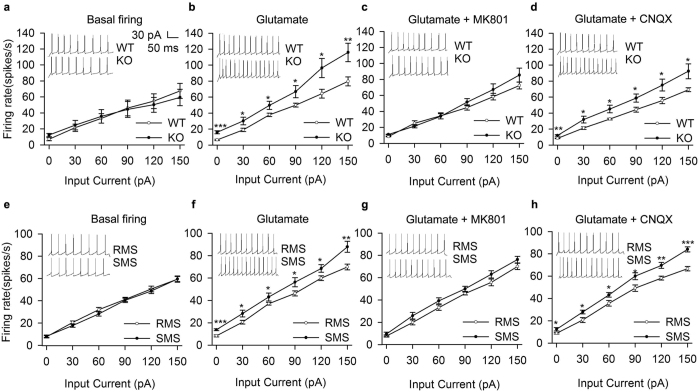
Electrophysiological study of swiprosin-1 on VN neuronal excitability *in vitro*. (**a**) Relationship between injected current and evoked firing rate of WT (open symbols) and KO (filled symbols) groups when no glutamate was applied. (**b**) Relationship between injected current and evoked firing rate of WT and KO groups during 30 μM glutamate was applied. (**c**) Relationship between injected current and evoked firing rate of WT and KO during 20 μM MK801 and 30 μM glutamate were applied. (**d**) Relationship between injected current and evoked firing rate of WT and KO upon application of 20 μM CNQX and 30 μM glutamate. (**e**) Relationship between injected current and evoked firing rate of RMS (open symbols) and SMS (filled symbols) groups when no glutamate was applied. (**f**) Relationship between injected current and evoked firing rate of RMS and SMS groups during 30 μM glutamate was applied. (**g**) Relationship between injected current and evoked firing rate of RMS and SMS groups upon application of 20 μM MK801 and 30 μM glutamate. (**h**) Relationship between injected current and evoked firing rate of RMS and SMS groups upon application of 20 μM CNQX and 30 μM glutamate. Representative traces of action potential elicited by 60 pA depolarizing currents. Data were quantified as the mean ± SEM. *p < 0.05, **p < 0.01, ***p < 0.001 compared to control or as indicated; n = 5–16 in each group.

To observe the effect of swiprosin-1 on neuronal excitability in the medial vestibular nucleus, the firing rate of neurons in swiprosin-1 knock out (KO) and wild type (WT) mice was recorded in brain slices in the presence or absence of glutamate. The results showed similar basal firing rates between the swiprosin-1 KO and WT mice (Fig. 7a). Upon incubation with 30 μM glutamate for 1 min, the firing frequency was higher in KO mice than in WT mice (Fig. 7b). Both MK801 and the α-amino-3-hydroxy-5-methyl-4-isoxazolepro- pionic acid (AMPA) receptor antagonist CNQX decreased the firing rate in the presence of glutamate but only MK801 eliminated the difference in excitability between KO and WT mice (Fig. 7c,d). We also noticed higher firing rates in cultured VN neurons of SMS mice than in RMS mice in the presence of glutamate (Fig. 7f) but not in the absence of glutamate (Fig. 7e). The difference between the RMS and SMS mice was eliminated by administration of MK801 but not CNQX (Fig. 7g,h).

## Discussion

Swiprosin-1 was differentially expressed in the brains of two mouse strains with varying MS sensitivities (SMS and RMS). Functional studies indicated that swiprosin-1 in the VN is a critical determinant of the susceptibility to MS.

An animal model with a spontaneous human disease is an ideal tool for studying the given disease. Currently, there are approximately 20 strains that simulate a number of human diseases, such as hypertension, diabetes and obesity in rodents, including in rats and mice^45^,^46^,^47^. These rodent models are typically obtained with classical inbreeding techniques after a directional screening for 10–20 generations^48^. Recent studies point to a strong genetic predisposition of individuals to MS^4^,^19^. Considering the possible links between genetic basis and MS susceptibility, we first established two mouse strains (SMS and RMS mice). The separation of susceptibility to MS into two stable strains took only 6 generations, confirming strong influence by genetic factors.

The huge variation in MS index in the natural population of mice as described above (Fig. 1a,b) poses great difficulty in screening anti-MS drugs. The generation of the SMS mouse strain represents an important advancement and provides a platform for drug screening and for basic research. For example, with the help of genomics, proteomics and metabonomics, it is possible to find the gene(s), protein(s) or metabolite(s) responsible for differences in susceptibility.

Swiprosin-1 is a newly discovered protein with few known functional implications. It is also referred to as EF-hand domain-containing protein D2 (EFhd2)^49^. In immune cells, swiprosin-1 was found to be regulated by calcium signalling^49^,^50^, protein kinase C^29^,^51^,^52^, and nuclear factor-κB^29^,^51^ and to be involved in apoptosis, actin bundling and polymerization^53^,^54^. It was also found to promote cell invasion and metastasis in cancer^55^. In the central nervous systems, swiprosin-1 was recently found in neurites and synapses and identified to affect microtubule transport in neurons and to play a significant role in Alzheimer’s disease and other neurological disorders^56^,^57^,^58^.

Proteomics, Western blot and RT-PCR analyses revealed lower expression of swiprosin-1 in SMS mouse brains than in RMS mouse brains. The significant down-regulation of swiprosin-1 at both the mRNA and protein levels in the VN of SMS mouse brains suggests that swiprosin-1 may be a genetic factor of susceptibility to MS. Although the concomitant down-regulation of swiprosin-1 mRNA and protein specifically in the VN of SMS mice suggests tissue-specific transcriptional mechanisms of swiprosin-1 gene regulation, additional post-translational mechanisms of swiprosin-1 regulation cannot be excluded. It also remains unclear whether the reduction in swiprosin-1 expression during MS is reversible, causative, compensatory or functionally irrelevant. However, our functional experiments indicated that swiprosin-1 is closely related to MS. The evidence supporting a specific relationship between swiprosin-1 and MS is summarized as follows: (1) Swiprosin-1 was detected at high levels in the cytoplasm of VN neurons, a brain region governing MS. (2) Swiprosin-1 levels in the VN decreased after rotation stimulus but were not changed by other stimuli, including hypoxia, hypothermia and heat. (3) Decreased swiprosin-1 levels after rotation stimulus required intact vestibular system. This decrease was abolished mice that were bilaterally labyrinthectomized with gentamicin. (4) Changes in swiprosin-1 levels in the VN are temporally associated with changes in the MS index. In addition, recovery of swiprosin-1 expression coincided with motor coordination recovery.

The decrease of swiprosin-1 after rotation is fairly rapid and does not seem to involve inhibition of gene expression. This notion was experimentally confirmed with cycloheximide, an inhibitor of protein synthesis: cycloheximide failed to prevent the decrease of swiprosin-1. In the contrary, decreased swiprosin-1 expression in response to rotation was prevented by bortezomib, which blocks proteasome-dependent protein degradation. We also found increased chymotrypsin-like protease activity after rotary stimulation, suggesting that the rapid decrease of swiprosin-1 is due to increased degradation and not reduced protein synthesis. This could also be supported by the identification of swiprosin-1 as a putative ubiquitin substrate^59^.

Interestingly, swiprosin-1 gene expression was changed in AD patients^33^,^57^, Parkinson’s patients^60^, schizophrenia patients^61^, and suicide victims^62^ and in a mouse model of amyotrophic lateral sclerosis/motor neuron disease^63^. Collectively, these data suggest that swiprosin-1 gene expression is regulated in neurodegenerative diseases and psychiatric disorders^58^. However, little is known regarding the emerging role of swiprosin-1 in neurological disorders. Therefore, further studies are crucial for determining the roles of swiprosin-1 in the pathophysiology of motion sickness and neurological disorders.

To examine the function of swiprosin-1 in MS, we conducted a set of experiments using three different techniques. All the results indicated that swiprosin-1 is an anti-MS protein. First, lentiviruses expressing siRNA of *swiprosin-1* (LV-shRNA-Swi) were used to down-regulate swiprosin-1 in the VN, and lentiviruses expressing full-length *swiprosin-1* (LV-Swi) were used to over-express swiprosin-1 in the VN. In wild-type control mice, LV-shRNA-Swi increased the MS index, and LV-Swi decreased the MS index. More interestingly, over-expressing swiprosin-1 in the VN of SMS mice decreased the MS index, whereas reducing swiprosin-1 in the VN of RMS mice increased the MS index. Second, mice lacking swiprosin-1 (*swiprosin-1*^−/−^ mice) were more sensitive to rotation in comparison to wild-type mice. Third, we conducted a set of electrophysiological experiments in brain slices. Glutamate is the main transmitter in the vestibular system. In microinjection experiments, the expression of swiprosin-1 was decreased by glutamate but not by histamine or acetylcholine. In addition, the NMDA receptor antagonist MK801 prevented the reduction in swiprosin-1 levels after rotation. Therefore, in this set of experiments, we selected glutamate as a stimulator of VN neuronal activity in brain slices to mimic the rotary stimulation in mice. The neuronal activity was enhanced by glutamate in brain slices obtained from either swiprosin-1 knockout mice or down-regulated mice (SMS) compared with their respective control. Evidently, the inhibition of swiprosin-1 on VN neuronal activity contributes to its anti-MS effect *in vivo*.

A recent study showed that deletion of the swiprosin-1 gene causes no detectable effect on brain anatomy or function^56^. Notably, vesicle transport velocity was enhanced in swiprosin-1 knockout primary hippocampal neurons and that swiprosin-1 inhibited microtubule gliding mediated by kinesin *in vitro*^56^. In recent studies, swiprosin-1 co-localized with neurite markers such as tau, microtubule-associated protein2 (MAP2), synapsin, and postsynaptic density 95 (PSD95)^57^, indicating that swiprosin-1 may play roles in both synapses and intracellular transport. The synaptic presence of swiprosin-1 was verified by biochemical analysis of isolated synaptosomes^56^,^57^. Mielenz *et al*. speculated that loss of swiprosin-1 could modulate long-term potentiation by modulating the delivery of kinesin-transported cargo and by orchestrating local actin dynamics in a dynamic equilibrium^64^. Given that swiprosin-1 is highly expressed in neurons of the VN and considering our results, it is possible that swiprosin-1 in VN neurons may play a role in synaptic plasticity and in the pathophysiology of motion sickness.

Habituation is a characteristic feature of MS. Desensitization has been reported to prevent MS in >85% of the subjects^65^. In our experiments, over-expressing swiprosin-1 in the VN shortened the adaptation time from 10 to 6 days. Consistent with this finding, habituation was not observed in the swiprosin-1 knockout mice.

Taken together, our data demonstrated that swiprosin-1 is a critical protein expressed in the VN that determined the susceptibility of mice to MS.

## Methods

### Animals

Mice (Kunming strain, outbred) weighing 18–22 g were provided by the Experimental Animal Center of the Second Military Medical University (Shanghai, China). During experiments, mice received humane care, and all mouse experiments were approved by the Second Military Medical University. All the experimental procedures were performed in accordance with the guidelines of the Second Military Medical University for the health and care of experimental animals.

A healthy adult male cynomolgus monkey weighing 3.5–4 kg was provided by the Suzhou Xishan Zhongke Drug R & D Co., Ltd. (Suzhou, China). All the experiments performed in the cynomolgus monkey were approved by the Science and Technology Department of Jiangsu Province. All the experimental procedures were performed in accordance with the guidelines of the Science and Technology Department of Jiangsu Province regarding the health and care of experimental animals. The monkey was fixed in a rotary monkey chair, deviated from the centre axis of rotation by approximately 60 cm. The rotational speed was 180°/s via clockwise rotation for 60 s, followed by emergency stops, and then counter-clockwise rotation for 60 s lasting a total of 60 min. The motion sickness symptoms in the cynomolgus monkey were scored according to evaluation criteria as described by Liu and Su60. Briefly, score = 1: lassitude (wryneck, eyes closed short), pale skin, licking lips, smacking, special position; score = 2: irritability, roaring, eyes closed more than 3 times in 1 min or eyes closed over 10 s for once; score = 4: urination, defecation; score = 8: nausea, retching; score = 16: vomiting. Scores were assessed six times and recorded at every 10-min interval. The highest score in one assessment was used if multiple symptoms occurred or if symptoms occurred several times. In addition, the final score was the sum of the six scores.

### Rotational stimulation

Rotational stimulation was performed according to the procedure described by Ossenkopp with minor modifications^66^. Each mouse was enclosed in an individual hyaline centrifuge cage (length = 15 cm, width = 15 cm, and height = 25 cm). The swing-arm on which the animal cage was suspended was mounted 60 cm from the axis of a turntable driven by a servo-controlled torque motor. The turntable had six swing-arms. The device began to rotate in the clockwise direction at a constant angular acceleration of 40°/s^2^. When the angular velocity reached 240°/s, was slowed at a constant angular deceleration of 40°/s^2^. Without pause, the device was rotated again in the counter-clockwise direction in the same manner. The swing frequency was 0.167 HZ, which was conducive to MS^67^,^68^. Mice were stimulated by rotating for 40 min and then placed on the ground for 5 min. MS symptoms were observed and recorded. The score of MS symptoms was obtained according to the previously depicted evaluation criteria^22^. This score, defined as the MS index, is suitable for mice.

### Lentiviral construction and preparation

The recombinant lentiviruses expressing the swiprosin-1 full-length coding sequence (LV-Swi) and the swiprosin-1 interference fragment (LV-shRNA-Swi) were produced by Shanghai Innovation Biotechnology Co. The viruses were concentrated by ultracentrifugation and titred by infection of confluent 293 T cells.

### Stereotactic injection of lentivirus targeting the swiprosin-1 protein in the VN

Stereotactic manipulations were performed in 15-week-old male mice to deliver 0.5 μL purified virus (2 × 10^10 ^Pfu/mL) LV-shRNA-Swi or LV-Swi to the VN at (−6.5 mm posterior and 1.1 mm lateral to the bregma and 5 mm below the skull surface^69^. After 3 days, rotary stimulation was performed, and the medulla oblongata was harvested according to the approved procedure. Correct positioning of the VN was verified by injecting methylene blue (1%) after the animals were sacrificed.

### Generation of swiprosin-1-deficient mice

Recombineering was used to generate a gene targeting construct from a bacterial artificial chromosome (BAC) containing the *swiprosin-1* gene (C57Bl/6 J inbred strain). E14 ES cells were transfected with this construct, and successfully targeted ES cells were injected into C57Bl/6 J blastocysts. Mice bearing this targeted allele of exon 2–4 deletion in the germline were bred to generate *swiprosin-1*^*−/−*^ mice.

### Two-dimensional fluorescence difference gel electrophoresis

Protein extracts were prepared from mouse brain tissues as described previously^70^. Proteins were labelled using a CyDye DIGE Fluor minimal labelling kit (GE Healthcare) according to the manufacturer’s instructions^71^. Briefly, Cy2 was used to label the pooled internal standard. Cy3 and Cy5 were used to randomly label the RMS and SMS samples. Following the labelling reaction, 50 μg each of Cy2, Cy3 and Cy5 labelled samples were mixed and diluted with rehydration buffer. For isoelectric focusing electrophoresis, the samples were placed in a strip holder using a step gradient protocol (30 v 6 hr, 60 v 6 hr, 200 v 1 hr, 500 v 1 hr, 1000 v 1 hr, and 8000 v 6 hr). After the IPG strips were equilibrated in SDS equilibration solution, and the proteins were further separated on 12.5% homogeneous SDS-PAGE gels (80 mV/gel for 20 min and 280 mV/gel for approximately 4 hr).

### Scanning and image analysis

Cy2, Cy3, and Cy5 labelled images for each gel were scanned using a Typhoon Trio variable imager (GE Amersham). The ratios of the log-standardized protein spot abundances (differences in expression) between the groups were computed (One-way ANOVA, p < 0.05). Differentially expressed protein spots were excised from the post-stained gel for further identification.

### Mass spectrometry and protein identification

The manually excised protein spots were digested with trypsin and then mixed with matrix solution for analysis using a 4700 MALDI-TOF/TOF Proteomics Analyzer (Applied Biosystems) according to the manufacturer’s instructions^72^.

### Immunostaining and blotting

The VN was dissected from mouse brainstems and homogenized in T–PER Tissue Protein Extraction Reagent (Pierce) supplemented with protease and phosphatase inhibitors (Merck). Samples were separated on an 8% SDS PAGE and transferred to nitrocellulose membranes (Pall Corporation). The membranes were blocked with 5% bovine serum albumin and blotted with antibodies. Anti-swiprosin-1 (IMGENEX) was used at a concentration of 1:2000, and anti-Tubulin (Beyotime) was used at 1:10000. Protein was visualized using IRDye-conjugated anti-mouse or anti-goat secondary antibodies at 1:5000. An Odyssey Infrared Imaging System (LI-COR) was used to analyses the results.

Each mouse was perfused with saline followed by formalin. The brains were fixed overnight at 4 °C in 4% paraformaldehyde and then treated in 30% sucrose at room temperature for 1 hr. The brains were embedded in paraffin and resectioned into 20-μm-thick coronal sections (bregma −0.8 mm to −8 mm). After deparaffination, endogenous peroxidase activity was blocked by incubating the sections in 1.5% peroxide in methanol for 20 min. The sections were blocked in PBS containing 2% normal goat serum, 1% BSA, and 0.3% Triton X-100 and incubated in anti-swiprosin-1 antibody (IMGENEX) at 1:200 overnight at 4 °C. Horseradish peroxidase (HRP)-labelled streptavidin and DAB were used to visualize each primary antibody.

### RNA isolation and quantitative RT-PCR

Total RNA was extracted from the brainstem of the mice in each group with Trizol reagent according to the manufacturer’s instructions. After reverse transcription, complementary DNA was used as templates for PCR. Primers for swiprosin-1 and an internal reference were as follows: swiprosin-1: forward, 5′-GTGTCCGTTGCTGTGTTGTG-3′, reverse, 5′-CCCCTCCGATTCTCATAGG T-3′; GAPDH: forward, 5′-GTATGACTCCACTCACGGCAAA-3′, reverse, 5′-GG TCTCGCTCCTGGAAGATG-3′. The housekeeping gene GAPDH was used as an internal control, and the amount of RNA was calculated by the comparative threshold cycle method as recommended by the manufacturer. Quantitative real-time PCR was carried out using an ABI 7500 real-time PCR system (Applied Biosystems, Foster, CA, USA).

### Climbing pole test

Mice were evaluated for their performance on a vertical pole (1 cm in diameter and 60 cm in height) after rotary stimulation. Each mouse was placed on a vertical pole and descended the pole with its head upward. The total time taken to descend three times was recorded.

### MS habituation

Animals were subjected to rotatory stimulation as described above at the same time every day for 2 weeks. The MS index was carefully recorded.

### Bilateral labyrinthectomy

Twenty male KM mice, each weighing 18–22 g, were used for this study. The animals were divided into two groups (n = 10 in each): I, control; II, Gentamicin (Zhong Xi Pharmaceutical Co., Ltd., Shanghai). Animals in group I received 50 μl saline in each tympanic cavity; animals in group II received 50 μl gentamicin (40 mg/ml) in each tympanic cavity. After inducing anaesthesia, the solutions were injected into the right middle ear through a 1-ml injector in the lower posterior part of the tympanic membrane. After each injection, animals were kept in the left lateral decubitus position for five minutes under mild anaesthesia to minimize reflux of the applied drugs. The left side of the ear received the same operation. Ten days later, the animals underwent rotatory stimulation and then were killed to obtain the labyrinth and vestibular nucleus. MS index and samples were discarded if the results of labyrinth HE staining indicated failed labyrinthectomy.

### Proteasome activity assay

Ice-cold PBS (20 μl) containing 5 mM EDTA (PBSE) was added to 1 mg VN tissue and sonicated on ice for 20 s with a pulse length of 1 s two times using a pulsed homogenizer (JY92-II DN, SCIENTZ, China). The obtained tissue lysates were centrifuged at 13,000 × *g* for 5 min at 4 °C. The supernatants were diluted to a concentration of 0.2 μg/μl total protein with ice-cold PBSE. Then, 50 μl corresponding to 10 μg total protein was added to 50 μl luminescent reagent containing the Ultra-Glo Luciferase and the signal peptide (Suc-LLVY-aminoluciferin) coupled to luciferin. After mixing the components and preincubating 60 min in a white 96-well plate at room temperature, the resulting luminescence was measured with an integration time of 1 s using a Multimode Reader (Infinite M200, TECAN) in luminometry mode^73^.

### Electrophysiology

Briefly, brains from 14- to 21-day-old mice were rapidly removed, and coronal brain slices containing the vestibular nuclei were cut using a vibrating blade microtome in ice-cold artificial cerebrospinal fluid that was bubbled continuously with 95% O_2_–5% CO_2_ and adjusted pH to 7.4. After 1.5 h recovery at room temperature, an individual slice was transferred to a submerged recording chamber and continuously superfused with oxygenated artificial cerebrospinal fluid at 24–26 °C at a rate of 3 ml/min. Slices were supplemented with bicuculline (20 μM) to block GABAergic neurotransmission. Neurons in the MVN were visualized with differential interference contrast optics under infrared illumination. Neurons were patched in whole-cell, current-clamp mode using an Axon clamp 700B amplifier. The membrane potential was sampled at 1 kHz to continuously measure the firing rate; 20–40 kHz sampling was used for all other measurements. Pipet and access resistances were bridge balanced throughout each experiment. Patch pipettes (4–8 MΩ) were filled with a solution containing the following: 135 mM potassium gluconate, 2 mM MgCl_2_, 0.1 mM CaCl_2_, 1 mM EGTA, 10 mM HEPES, and 2 mM Na2ATP (pH 7.2, 285 mOsm).

### Statistical Methods

The data were expressed as the mean ± SD or mean ± SEM. Comparisons of parameters among two groups were made by unpaired Student’s t-tests. Data involving more than two groups were assessed by analysis of variance (ANOVA).

## Additional Information

**How to cite this article**: Wang, Z.-B. *et al*. Low level of swiprosin-1/EFhd2 in vestibular nuclei of spontaneously hypersensitive motion sickness mice. *Sci. Rep.* **7**, 40986; doi: 10.1038/srep40986 (2017).

**Publisher's note:** Springer Nature remains neutral with regard to jurisdictional claims in published maps and institutional affiliations.

## Supplementary Material

Supplementary Information
