# Supplementary material for: Low level of swiprosin-1/EFhd2 in vestibular nuclei of spontaneously hypersensitive motion sickness mice
Source: Sci Rep. 2017 Jan 27;7:40986. doi: 10.1038/srep40986 (PMC5269593; doi:10.1038/srep40986)
Supplement: Supplementary Information [file srep40986-s1.doc]

**Low level of swiprosin-1/EFhd2 in vestibular nuclei of spontaneously hypersensitive motion sickness mice**

Zhi-Bin Wang^1^, Ping Han^1^, Ling-Chang Tong^1^, Yi Luo^2^, Wei-Heng Su^3^, Xin Wei^1^, Xu-Hong Yu^1^, Wei-Ye Liu^1^, Xiu-Hua Zhang^1^, Hong Lei^1^, Zhen-Zhen Li^1^, Fang Wang^2^, Jian-Guo Chen^2^, Tong-Hui Ma^3^, Ding-Feng Su^1^, Ling Li^1^

^1^ Department of Pharmacology, College of Pharmacy, Second Military Medical University, Shanghai 200433, China

^2^ Department of Pharmacology, Tongji Medical College, Huazhong University of Science and Technology, Wuhan, Hubei 430030, China

^3^ Basal medical College, Dalian Medical University, Dalian, Liaoning 130041, China

The first three authors contributed equally to this work

Correspondence should be addressed to Ling Li (lingli_z163@163.com), Ding-Feng Su (dfsu2008@gmail.com), Jian-Guo Chen (chenjg0428@hotmail.com) or Tong-Hui Ma (Math108@nenu.edu.cn).

**Supplement 1**

1780pmf


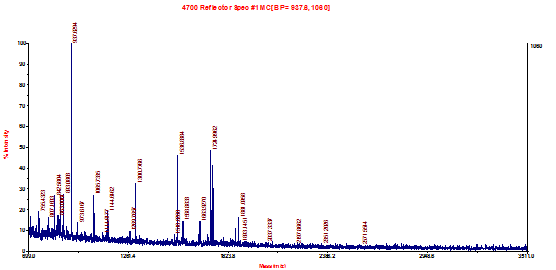


msms

The matched peptides were shown in bold red.

**1** MATDELASKL SRRLQMEGEG GEATEQPGLN GAAAAAAAEA PDETAQALGS

**51** ADDELSAKLL R**RADLNQGIG EPQSPSRRVF NPYTEFKEFS R**KQIKDMEKM

**101** FKQYDAGRDG FIDLMELKLM MEKLGAPQTH LGLKSMIQEV DEDFDSKLSF

**151** R**EFLLIFRK**A AAGELQEDSG LQVLARLSEI DVSTEGVKGA KNFFEAKVQA

**201** INVSSR**FEEE IKAEQEER**KK QAEEVKQRKA AFKELQSTFK

The mass information used to identify swiprosin-1 was showed.

**Supplement 2**


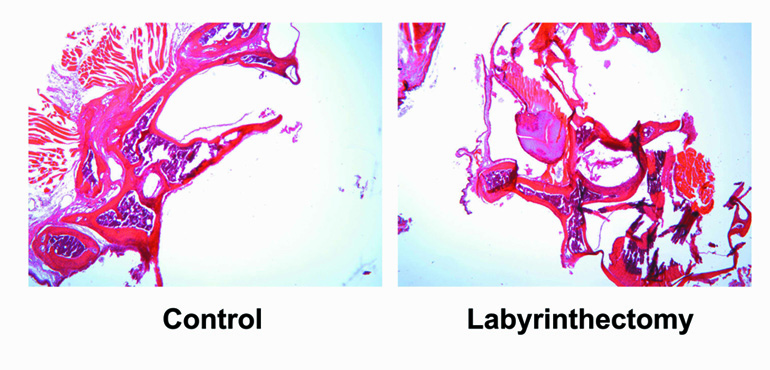


Histological results showed that hair cells and sertoli cells were practically absent in mice receiving gentamicin caused labyrinthectomy.

**Supplement 3**

**
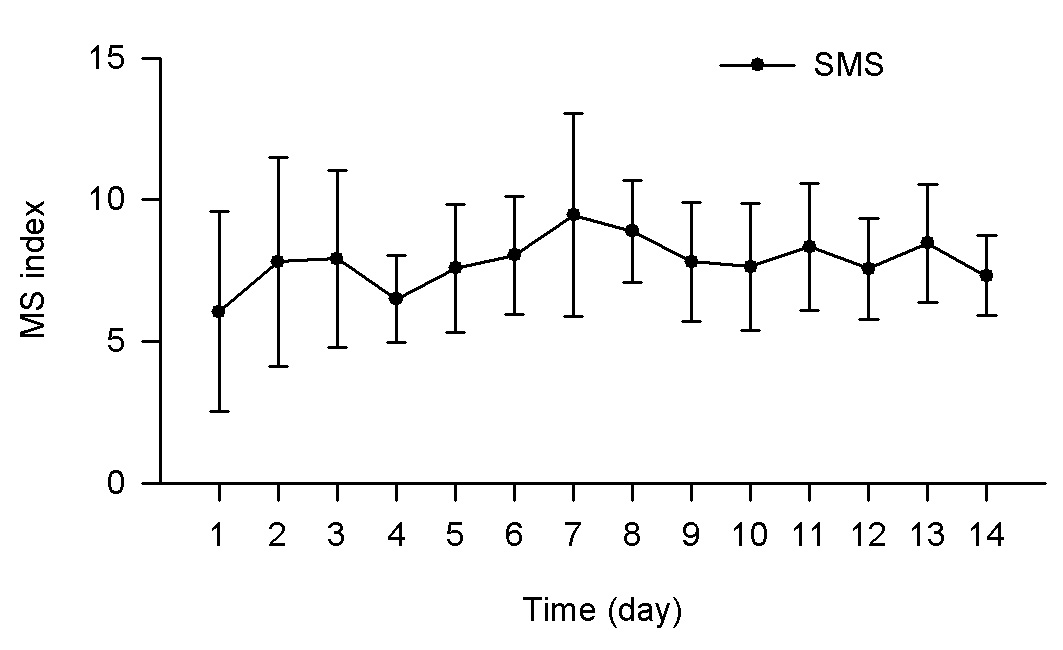
**

Habituation behavior disappeared in SMS mice. Data are quantified as mean ± SD, n=7.

**Supplement 4**


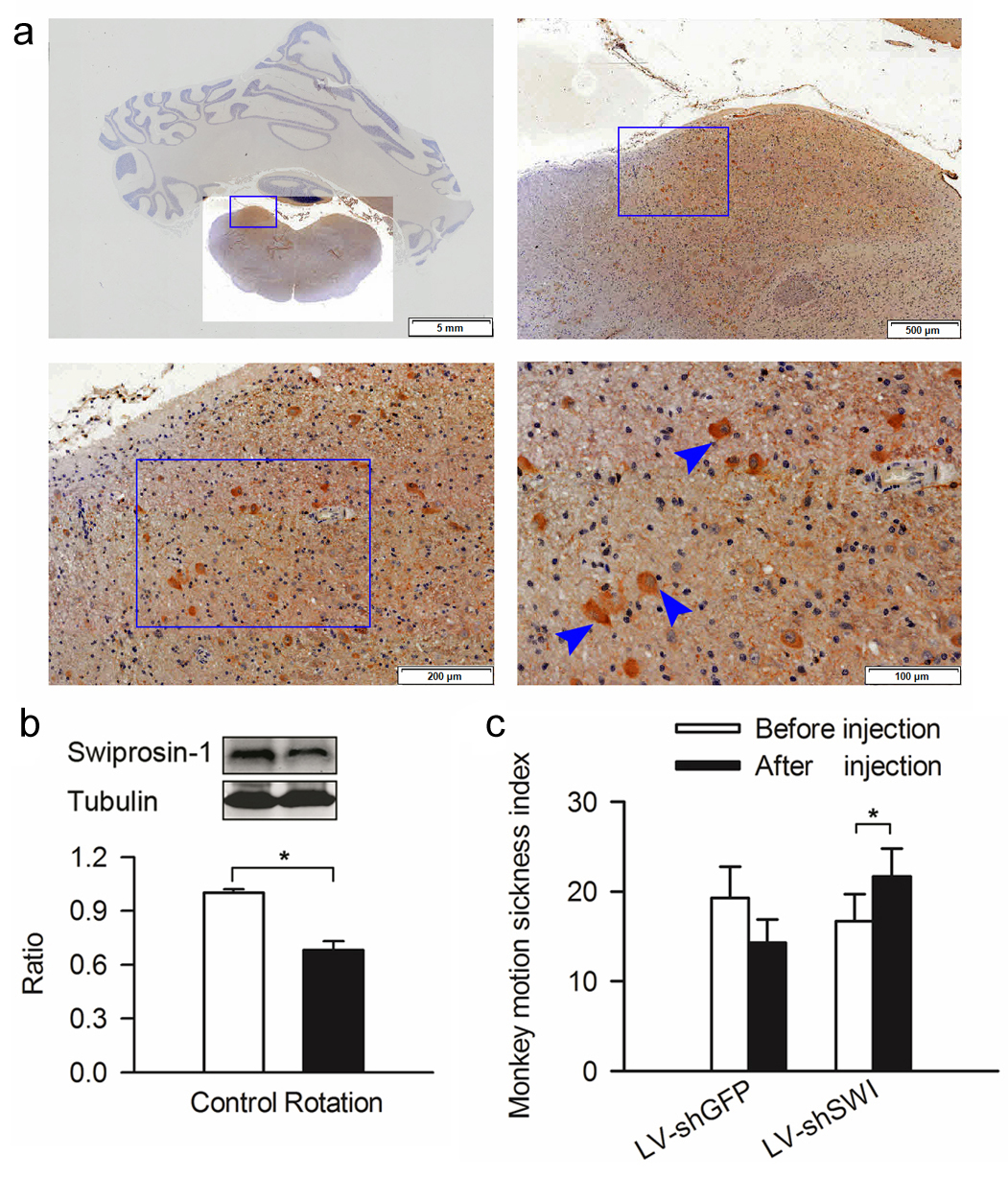


**Verify the distribution and function of swiprosin-1 on cynomolgus monkey.** (**A**) Localization of swiprosin-1 in the VN of cynomolgus monkey medulla oblongata by immunohistochemistry. (**B**) Protein levels of swiprosin-1 were determined by Western blotting of the medulla oblongata from cynomolgus monkey treated with rotary stimulation. **(C)** MS index after injection of LV-shGFP (control group) or LV-shSWI (RNAi group) in the VN of cynomolgus monkey. Data quantified as mean ± SD. *p<0.05 compared as indicated; n=3 in each group.
